# Metabolomic and Transcriptomic Analyses Reveal the Molecular Mechanism of Flower Color Variations in *Rosa chinensis* Cultivar ‘Rainbow’s End’

**DOI:** 10.3390/metabo16010032

**Published:** 2025-12-27

**Authors:** Junfei Sun, Fengshan Ren, Xianshui Meng, Guizhi Dong, Xiaohong Zhang, Yi Li

**Affiliations:** 1 Institute of Leisure Agriculture, Shandong Academy of Agricultural Sciences, Jinan 250100, China; sunjunfei@saas.ac.cn (J.S.); renfengshan@saas.ac.cn (F.R.); 2 Pingyin County Natural Resources Bureau, Jinan 250400, China; xianshuimeng@126.com (X.M.); dongguizhi8888@163.com (G.D.); falv0922@163.com (X.Z.)

**Keywords:** anthocyanin, carotenoid, flower color, RNA-seq

## Abstract

Background: *Rosa chinensis,* commonly known as the Chinese rose, is one of the most economically significant ornamental plants worldwide. The *Rosa chinensis* cultivar ‘Rainbow’s End’ notably transitions in color from yellow to red throughout its blooming phase; however, the chemical and molecular foundations underlying this floral color transformation remain inadequately understood. Methods: This study used the petals of the Rosa ‘Rainbow’s End’ cultivar at four developmental stages (R1, R2, R3, and R4) for targeted metabolomic and transcriptomic analyses. Results: Targeted metabolomic analyses revealed that the majority of anthocyanidin metabolites were highest at stages R2 and R3 and lowest at R1 and R4. In contrast, most carotenoid metabolites reached their highest levels at R1 and declined continuously from R2 to R4. These results were consistent with the color phenotype of Rosa ‘Rainbow’s End’ petals and suggested that both anthocyanins and carotenoids play critical roles in flower color variation. Specifically, an upregulation of *CHS*, *ANS*, and *UGT* genes in the anthocyanin biosynthesis pathway was observed in R2 and R3, coinciding with the expression of two MYB transcription factors (MYB14 and MYB54). Conversely, consistent downregulation of *PSY*, *PDS*, *Z-ISO*, *ZDS*, *CHYB*, and *NCED* genes in the carotenoid biosynthesis pathway was detected in R2 to R4 and was associated with four MYB transcription factors (MYB20, MYB43, MYB44, and MYB86). Conclusions: Rosa ‘Rainbow’s End’ is an excellent model for studying variations in flower color. The expression patterns of the identified structural genes involved in anthocyanin and carotenoid biosynthesis pathways, along with the related MYB transcription factors, were aligned with the levels of metabolite changes in the petals of four flowering stages. These genes and transcription factors are likely responsible for the color shifts in Rosa ‘Rainbow’s End’. This study clarifies the mechanisms underlying color changes in Rosa ‘Rainbow’s End’ and provides a theoretical basis for future flower breeding efforts.

## 1. Introduction

Floral coloration is a key economic trait in floricultural crops, displaying a wide color range from red to purple. The main natural pigments are flavonoids, carotenoids, betalains, and alkaloids [1]. The flavonoid/anthocyanin biosynthesis pathway is regulated by conserved structural genes such as phenylalanine ammonia-lyase (*PAL*), chalcone synthase (*CHS*), chalcone isomerase (*CHI*), flavanone 3-hydroxylase (*F3H*), dihydroflavonol-4 reductase (*DFR*), anthocyanin synthase (*ANS*), and UDP-glycosyltransferase (*UGT*). These enzymes catalyze reactions that affect aglycone structures and glycosylation, producing red, purple, and blue colors [2,3,4,5]. Similarly, the carotenoid pathway, which involves genes like phytoene synthase (*PSY*), phytoene desaturase (*PDS*), 15-cis-ζ-carotene isomerase (*Z-IOS*), ζ-carotene desaturase (*ZDS*), lycopene β-cyclase (*LCYB*), β-carotene hydroxylases (*CHYB*), zeaxanthin epoxidase (*ZEP*), and 9-cis-epoxy carotenoid dioxygenase (*NCED*), governs the yellow-to-red color spectrum [6,7,8]. These pathways are regulated by transcription factors: the MYB bHLH WD40 complex (MBW) promotes *DFR* and *ANS* gene expression [9,10,11], while repressive MYBs (like MYB27) compete for bHLH partners to slow the pathway [12,13]. MYB1 influences both rose anthocyanin biosynthesis and carotenoid metabolism [14,15]. Flowers like Calendula officinalis can synthesize unique carotenoids, such as lycopene, which are found only in orange to red petals [16,17,18], producing diverse petal colors. Transcriptomic studies have shown that these pathways are sequentially regulated during color transitions; during the carotenoid phase, anthocyanin genes (*CHS*, *DFR*, and *ANS*) are suppressed [19]. As the color shifted, anthocyanin biosynthesis increased, while carotenoid gene expression (*PSY* and *PDS*) was downregulated, indicating a pathway shift rather than just substrate competition. Environmental factors (light, temperature, ethylene) and hormones (ABA, JA, SA) influence this via transcription factors (ERFs, NAC, WRKY, MADS-box), regulating MBW activity to alter color speed with seasons or growth conditions [20,21,22,23,24,25].

*Rosa chinensis* is a key ornamental plant with diverse flower colors and high market value [26,27]. Recently, “color-changing” cultivars, whose flowers shift from pale-greenish-white or yellow at anthesis to pink, red, or purple, have attracted horticultural and scientific interest [28]. Unlike traditional varieties, these roses produce both anthocyanins and carotenoids while precisely switching pigment dominance, making them ideal for studying pigment regulation and color changes. Few studies have captured high-resolution data on metabolites and transcripts in the same floral tissues during this period.

In this study, we used the *Rosa chinensis* cultivar, Rainbow’s End’, which is known for its distinct petal color changes during development. By combining targeted metabolomics and RNA-seq across four developmental stages, we integrated omics approaches to explore the interactions between structural genes and regulators that control pigment transitions, aiming to reveal the molecular mechanism of flower color changes during the flowering process of Rosa ‘Rainbow’s End’. Our results address a knowledge gap in ornamental color biology and identify targets for breeding flowers with specific colors.

## 2. Materials and Methods

### 2.1. Plant Materials

Rosa ‘Rainbow’s End’ was obtained from the Jiyang Base, Experimental Demonstration Base of Shandong Academy of Agricultural Sciences (E: 116°59′0.43″, N: 36°58′31.62″, China). In June 2024, fresh petals at each stage were randomly selected and collected in the afternoon, rapidly frozen with liquid nitrogen, and stored at −80 °C. This study included three biological experiments, each using petal tissues from three different specimens. Spectrophotometry was used to measure anthocyanin characteristics during peak flower maturity.

### 2.2. Pigment Separation

To identify the floral pigments, 50 mg of flower petal tissue was ground in liquid nitrogen. The samples were dissolved in 200 µL methanol, followed by the addition of 200 µL water and 200 µL dichloromethane for phase separation. The mixture was centrifuged at 13,000 rpm for 5 min, and the pigment layers were observed: anthocyanins in the upper aqueous layer and carotenoids in the lower organic layer [29,30].

### 2.3. Targeted Metabolome Profiling and Analysis

Anthocyanins and carotenoids were analyzed using a scheduled multiple reaction monitoring (MRM) method. For this purpose, experiments were carried out with a triple quadrupole-linear ion trap mass spectrometer, specifically the QTRAP^®^ 6500+ LC-MS/MS System from Sciex (Shanghai, China), which conducted both linear ion trap and triple-quadrupole scans. Data acquisition was conducted using Analyst 1.6.3 software, and all metabolite quantifications were performed using Multiquant 3.0.3 software, both of which were products of Sciex. Mass spectrometer parameters were optimized with great attention, including the declustering potentials (DP) and collision energies (CE) for each MRM transition. Each phase had a unique set of MRM transitions, which were recorded based on the metabolites released during that time [31,32].

### 2.4. RNA-Seq

The RNA samples from the four different blooming periods of ‘Rainbows End’ were then processed with both PacBio Iso-Seq and Illumina sequencing services, performed by Qingdao Standard Testing Co., Ltd. in Qingdao, China. Total RNA was extracted from four distinct blooming periods of the ‘Rainbows End’ variety, following the manufacturer’s instructions for the Omega plant RNA extraction kit (Norcross, GA, USA). The mRNA was stored in an ultra-low temperature freezer at −80 °C.

After verifying the quality of the sample, the next steps in the library construction process were as follows. (1) Eukaryotic mRNA was enriched by magnetic beads conjugated to Oligo (dT). (2) A fragmentation buffer was added to randomly shear the mRNA. (3) The fragmented mRNA was used as the starting material to produce both the first and second strands of complementary DNA, and the resulting cDNA was purified. (4) Once purified, double-stranded cDNA was obtained, and terminal repair was performed. First, an adenine base was added to the end of the cDNA, and then the ligation of sequencing adapters occurred. Finally, the fragments were size-selected using AMPure XP beads (Beckman Coulter, Brea, GA, USA). (5) The cDNA library was completed by PCR amplification. After completing the above steps, the constructed library underwent a preliminary quality assessment. First, a Qubit 3.0 fluorometer (Thermofisher, Waltham, MA, USA) was used for initial quantification to confirm that the concentration met the requirement of no less than 1 ng/ul. The inserted fragments in the library were analyzed using the Qsep400 (Bioptic, New Taipei, China) high-throughput analysis system. If the fragments were found to be satisfactory, qPCR (Q-PCR) was used to measure the effective concentration of the library (>2 nM) to ensure that the library was intact. Once the quality assessment was completed, the prepared library was sequenced on the Illumina NovaSeq6000 (San Diego, CA, USA) platform in PE150 read mode. To enable this process, the designated reference genome acted as the main template for aligning the sequences and performing subsequent analytical tasks. The reference genome used was *Rosa chinensis* GCF_0034745.2. Genome.fa. Sci Tech used the HISAT2 [33] program to efficiently and precisely align Clean Reads with the reference genome. The processed reads were then assembled using StringTie v2.2.3 [34] to reconstruct the transcriptome for further analysis.

Transcript or gene expression levels were calculated using StringTie, which employs FPKM [35] for normalization using a maximum likelihood estimation approach. After preprocessing and normalization, differential expression analysis was performed using DESeq2 [36], and EBSeq was used as an alternative method when there were no biological replicates in the groups. When identifying differentially expressed genes, we applied a threshold of fold change ≥ 1.5 and a False Discovery Rate (FDR) < 0.01 for filtering. The fold change, which quantifies the ratio of expression levels between two samples or groups, and the false discovery rate (FDR), a statistical measure that adjusts *p*-values to account for multiple comparisons, were both critical metrics for assessing the significance of observed expression differences. For comparison, the logarithm of the fold-change (log2FC) was calculated. Differentially expressed genes (DEGs) were identified using the criteria of log2FC ≥ 2 and *p*-value ≤ 0.05.

### 2.5. qRT-PCR

The Applied Biosystems StepOnePlus Real-Time PCR System (Thermo Fisher, Waltham, MA, USA) was used to perform qRT-PCR. TaKaRa TB Green 5 Premix Ex Taq 5 (Tli RnaseH Plus) was used as the reagent for the reactions. qRT-PCR analysis used specific primers, which are listed in Table S6.

## 3. Result

**Figure 1:**
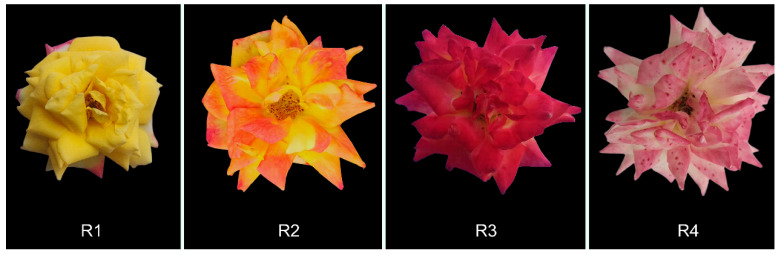
Stages of Rosa ‘Rainbow’s End’ flowering. (**R1**), the first date of petal sampling, the flower was fully open, and the petals were yellow; (**R2**), 4 days after R1, with orange-yellow petals; (**R3**), 8 days after R1, and the petals turned fully red; (**R4**), 12 days after R1, with white-pink petals.

We analyzed the metabolomic and transcriptomic profiles of Rosa’ Rainbow’s End across four flowering stages (Figure 1). Four stages of Rosa ‘Rainbow’s End’ flowering were distinguished based on time after flower opening and flower color: fully yellow flowers (R1), yellow-orange flowers (R2), fully red flowers (R3), and white-pink flowers (R4). A comparative analysis explored anthocyanin and carotenoid biosynthesis A comparative analysis explored anthocyanin and carotenoid biosynthesis.

### 3.1. Analysis of Targeted Anthocyanin and Carotenoid Metabolites in Flowers of Various Colors

**Figure 2:**
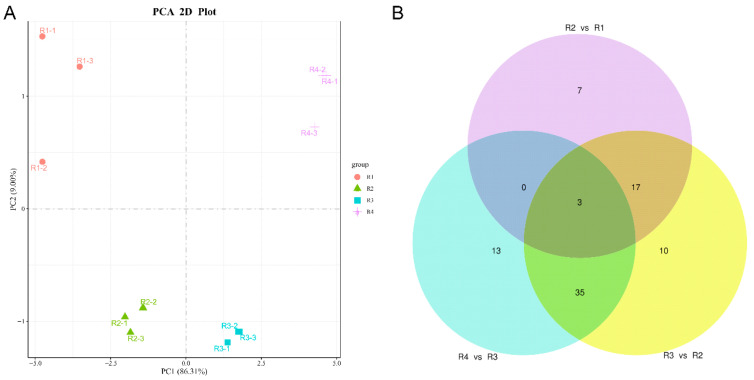
(**A**) PCA of the metabolites. The PCA map has an *x*-axis that shows PC1 and a *y*-axis that shows PC2. (**B**) Venn diagram showing the overlaps and unique features among the three sets.

We built a metabolite library using UPLC/MS and performed principal component analysis (PCA) (Figure 2A) in our analysis of Rosa ‘Rainbow’s End’ petals. The primary component (PC1) accounted for 86.31% of the variance, and the second component (PC2) explained 9.00%. This analysis revealed a clear separation across the four flowering stages, indicating a distinct trend.

Pairwise comparisons of Rosa ’Rainbow’s End’ petal colors were systematically performed, and samples were divided into three comparisons: R2 vs. R1, R3 vs. R2, and R4 vs. R3. Three metabolites were detected in all comparisons (Figure 2B). The connections between matches indicated the DEMs that existed in both comparisons, and the center showed three metabolites that appeared in all comparisons in the Venn diagram. Changes in the metabolites of both anthocyanidins and carotenoids were considered key drivers of petal color changes over time.

**Figure 3:**
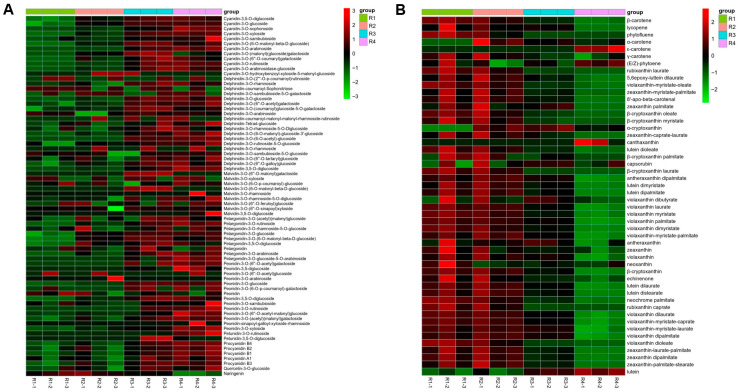
(**A**) Analysis of differentially accumulated anthocyanins across the four stages. (**B**) Analysis of differentially accumulated carotenoids across the four stages. Standardized parameters were used in log2. Red indicates a high number of metabolites, and green indicates a low number.

When comparing R2 and R1, we found 24 metabolites that were differentially expressed and linked to anthocyanin biosynthesis, which can be categorized into five major subclasses: 11 cyanidin, four delphinidin, one malvidin, six pelargonidin, and two peonidin. Specifically, among these DEMs, more than 21 showed increased accumulation in R2, while three showed reduced levels (Figure 3A and Table S1a). Moreover, three DEMs with differential accumulation were identified in the carotenoid metabolic pathway. These metabolites were divided into two primary categories: two carotenes and one xanthophyll. Among these metabolites, two were found to have higher levels than those in R2, while the abundance of another metabolite decreased (Figure 3B and Table S2a).

When comparing R3 and R2, three differentially expressed metabolites involved in anthocyanin biosynthesis were found, which could be divided into six primary groups: 1 cyanidin, six delphinidin, three malvidin, five pelargonidin, six peonidin, and two petunidin. Approximately 32 anthocyanin DEMs increased in R3, with only one showing a decreased abundance (Table S1b). Conversely, 32 DEMs involved in carotenoid metabolism were differentially accumulated, falling into two main categories: four carotenes and 28 xanthophylls. All DEMs in this comparison exhibited a decline in abundance in R3 (Table S2b).

The R4 vs. R3 comparison identified 12 differentially accumulated DEMs pertinent to anthocyanin-targeted metabolism, distributed into six major categories: two cyanidin, two delphinidin, three malvidin, one pelargonidin, two peonidin, and two petunidin. Among these, six DEMs showed increased abundance in R4, while the remaining six DEMs experienced a decline in abundance (Table S1c). In contrast, 39 DEMs were involved in carotenoid metabolism, which were also sorted into two main categories: three carotenes and 36 xanthophylls. Among these, two DEMs showed an increase in abundance in R4, whereas 37 showed a decrease (Table S2c).

We also analyzed changes in anthocyanins and carotenoids across three additional comparison groups (R3 vs. R1, R4 vs. R1, and R4 vs. R2) to complement our findings. In R3 vs. R1, we identified 38 anthocyanidin DEMs, of which 37 increased in R3, and one decreased; we also found 30 carotenoid DEMs, of which three increased, and 27 decreased in R3. In R4 vs. R1, 41 anthocyanidin DEMs were detected, of which 38 were increased, and three were decreased in R4. In addition, 46 carotenoid DEMs were detected, of which five were increased, and 41 were decreased in the R4 group. In R4 vs. R2, 33 anthocyanidin DEMs were observed, of which 31 increased, and two decreased in R4. Meanwhile, 43 carotenoid DEMs were identified, of which three increased, and 40 decreased in the R4 group. These results are presented in Tables S3 and S4, which align with the color phenotypes observed in Rosa ‘Rainbow’s End’.

In addition, the ratios of each anthocyanidin and carotenoid metabolite class in the total amount during the four flowering stages of Rosa ’Rainbow’s End’ are presented in Table 1. The total amount of cyanidin metabolites was significantly higher than that of other anthocyanins at all stages of the flower petals (Table S1a–c). This result was consistent with the red coloration of Rosa ‘Rainbow’s End’ flowers. In contrast, the total amount of violaxanthin metabolites belonging to xanthophyll was significantly higher than that of other carotenoids (Table S2a–c). This result was consistent with the yellow color fading of Rosa ‘Rainbow’s End’.

### 3.2. Differentially Expressed Genes (DEGs) Analysis

**Figure 4:**
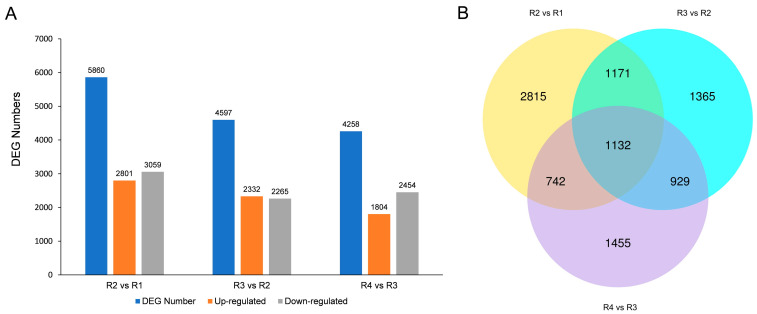
DEGs expression analysis. (**A**) Numbers of upregulated and downregulated DEGs in different comparisons. (**B**) Venn diagram of DEGs in different control groups.

To gain insights into the gene expression profile of Rosa ‘Rainbow’s End’ at different flowering stages, we conducted RNA-seq analysis. After quality filtration, the sequences exhibited remarkable integrity, with an average Q30 score of 97.13%. The mean GC content was 45.79% (Table S5). The differential gene expression analysis employed criteria where |log2| was greater than 1 and the significance level was set at 0.005 to find out differentially expressed genes. The highest number of such genes was identified in the comparison between R2 and R1 (5860 genes). This was followed by a comparison between R3 and R2, which yielded 4597 genes. A comparison between R4 and R3 revealed the fewest differentially expressed genes, numbering 4258 (Figure 4A). Notably, 1132 unigenes were consistently identified as differentially expressed genes across all group comparisons (Figure 4B).

There were more downregulated genes than upregulated genes in R2 vs. R1 and R4 vs. R3; however, there were slightly more upregulated genes than downregulated genes in R3 vs. R2. The total number of DEGs decreases continuously with the opening time of flowers

### 3.3. Gene Ontology (GO) Analysis of DEGs

**Figure 5:**
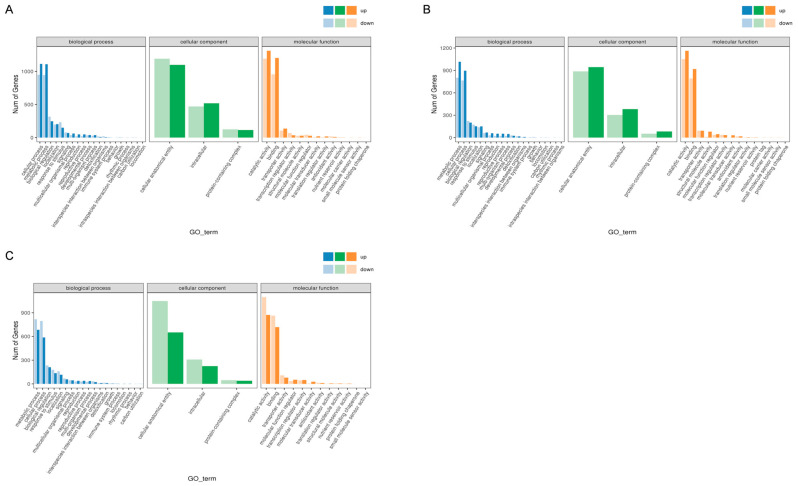
GO enrichment diagram of differentially expressed genes in the three comparison groups. (**A**) R2 vs. R1, (**B**) R3 vs. R2, and (**C**) R4 vs. R3.

GO is widely used in bioinformatics to classify gene functions into three main areas: Biological Process (BP), Cellular Component (CC), and Molecular Function (MF). An analysis of the top 20 most significantly enriched GO terms was performed for each group after the GO enrichment analysis (Figure 5).

In the R2 versus R1 comparison, most of the significant enrichments were found in the “cellular process” and “metabolic process” categories of BP, including 2063 and 2053 DEGs, respectively. A large proportion of DEGs were linked to the “cellular anatomical entity” category in CC, totaling 2292 DEGs. Regarding the MF category, most DEGs were classified as “catalytic activity” and “binding,” with 2505 and 2053 DEGs, respectively.

For the R3 versus R2 comparison, “metabolic process” and “cellular process” remained the main categories within BP, with 1816 and 1661 DEGs, respectively identified. At the same time, “cellular anatomical objects” was the most representative category of CC, with 1831 DEGs. In the MF process, the two main functional categories with the most differentially expressed genes were “catalytic activity” and “binding,” accounting for 2214 and 1711 DEGs.

Compared to R4 and R3, “metabolic process” and “cellular process” were the upper categories in BP at 1503 and 1383 DEGs, respectively, with most DEGs corresponding to “cellular anatomical objects” in the CC category, with a total of 1699 DEGs. Within the MF category, “catalytic activity” and “binding” included the largest numbers of DEGs, with 1971 and 1581 DEGs, respectively.

### 3.4. Kyoto Encyclopedia of Genes and Genomes (KEGG) Analysis of DEGs

**Figure 6:**
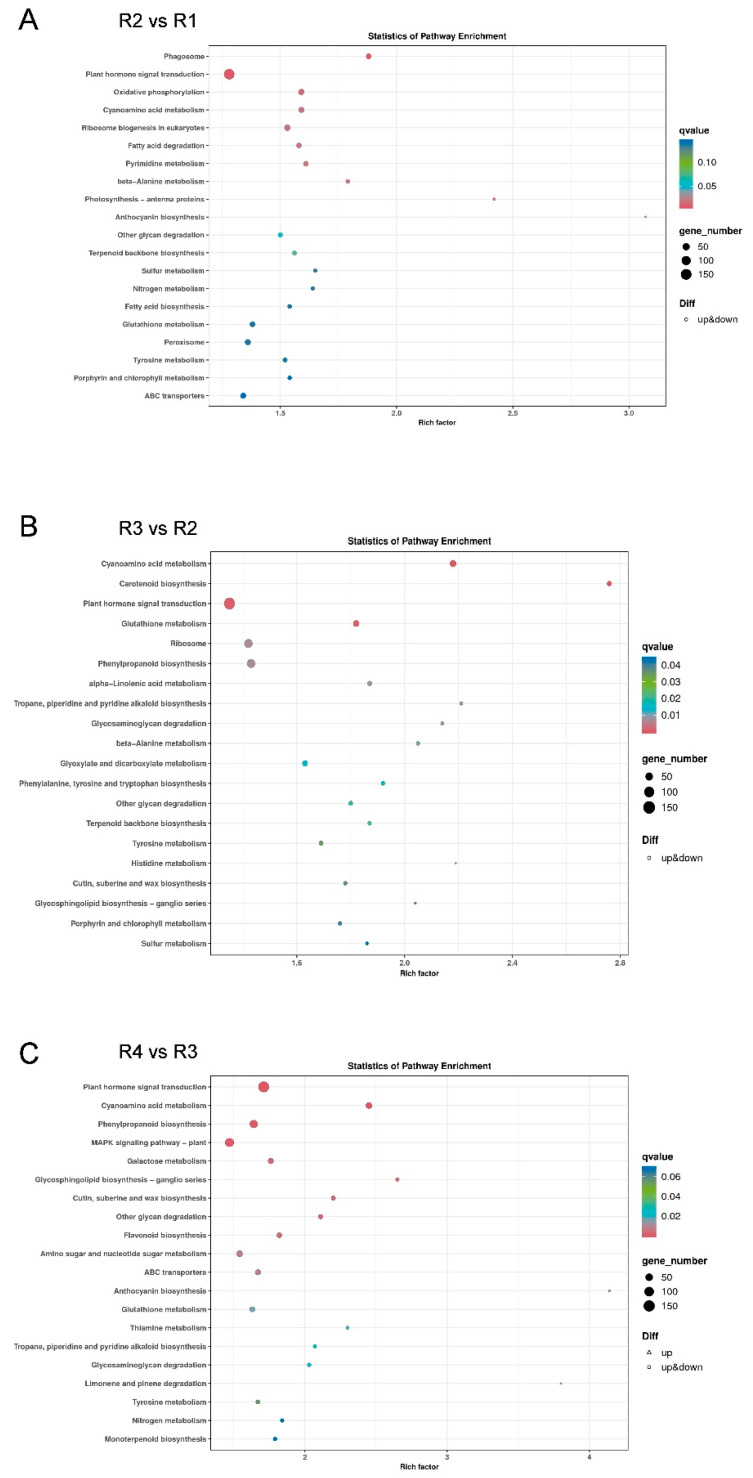
KEGG enrichment bubble diagram of differentially expressed genes in the three comparison groups. (**A**) R2 vs. R1, (**B**) R3 vs. R2, and (**C**) R4 vs. R3.

The analysis identified the top 20 most significantly enriched KEGG pathways in each group (Figure 6). The bubble chart visualizes these pathways enriched by DEGs in the comparisons of R2 vs. R1, R3 vs. R2, and R4 vs. R3. It is important to note that genes related to ‘Anthocyanin biosynthesis’ and ‘Carotenoid biosynthesis’ pathways might have an impact on the biosynthesis and accumulation of anthocyanins and carotenoids in the petals of Rosa ‘Rainbow’s End.’

When comparing R2 and R1, the anthocyanin biosynthesis pathway was found to have 11 genes, among which six were differentially expressed: four were upregulated, and two were downregulated. In the carotenoid biosynthesis pathway, 66 genes were identified when comparing R3 and R2, 27 of which were DEGs: seven were upregulated, and 20 were downregulated. Conversely, in the R4 vs. R3 comparison, it was found that the anthocyanin biosynthesis pathway had 11 genes, 6 of which were DEGs—one showed upregulation, and five showed downregulation.

These findings indicate that the anthocyanin biosynthesis pathway was significantly activated in the R2 vs. R1 and R4 vs. R3 comparisons, with most DEGs showing upregulation in the R2 vs. R1 group and downregulation in the R4 vs. R3 group. Conversely, in the R3 vs. R2 comparison, the carotenoid biosynthesis pathway was significantly enriched, with most DEGs being downregulated. This indicates that carotenoid synthesis was active during the initial stages of flowering in Rosa’ Rainbow’s End, but as flowering progressed to the middle stage, it became repressed, and subsequently, the anthocyanin biosynthesis pathway was active at the middle stage but decreased in the late stages.

### 3.5. Transcription Factors (TFs) Analysis

**Figure 7:**
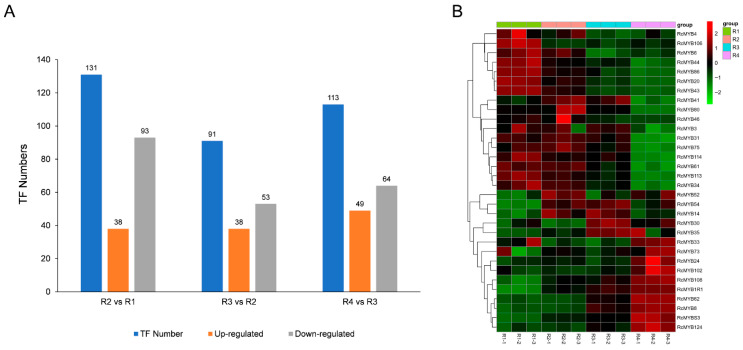
Transcription factor analysis. (**A**) Upregulation and downregulation of differentially expressed genes. Expressed TFs in different comparisons. (**B**) Heatmap of MYB family member expression. Standardized parameters were used in log2. Red indicates a high expression level, and green indicates a low expression level.

This study identified TFs showing differential gene expression in the RNA-sequencing data (Figure 7A). A comparison between R2 and R1 revealed 131 TFs with significantly differential expression, including 38 upregulated and 93 downregulated in R2. For the R3 vs. R2 comparison, 91 TFs showed significant differential expression; among these, 38 were upregulated, and 53 were downregulated in R3. The R4 vs. R3 comparison identified 113 TFs with significant differential expression: 49 upregulated and 64 downregulated in R4.

Given the importance of the MYB transcription factor family in floral coloration, we annotated all MYB TFs and identified 33 members. An expression heatmap was created using transcriptomic data (Figure 7B). The results showed that four MYB genes were highly expressed in R1, then decreased in R2, and remained low in R3 and R4. These genes, including *RcMYB20* (gene-LOC112191352), *RcMYB43* (gene-LOC112172176), *RcMYB44* (gene-LOC112169043), and *RcMYB86* (gene-LOC112175776), may be associated with carotenoid metabolic processes in Rosa ‘Rainbow’s End’. Conversely, two MYB genes, *RcMYB14* (gene-LOC112197815) and *RcMYB54* (gene-LOC112179065), showed higher expression in R2 and R3 but lower expression in R1 and R4, indicating their possible roles in anthocyanin biosynthesis and the red coloration of Rosa ‘Rainbow’s End’.

### 3.6. Differential Expression Gene Screening in the Anthocyanin and Carotenoid Biosynthetic Pathway

**Figure 8:**
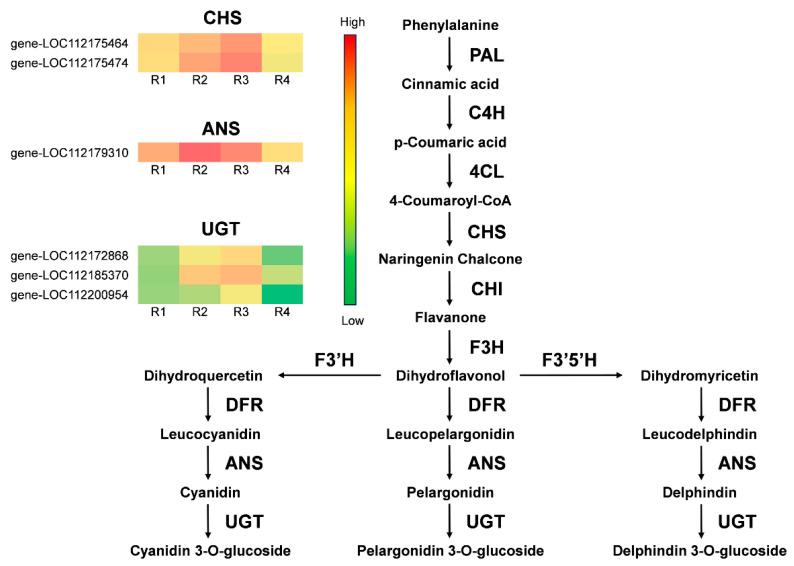
Expression patterns of key anthocyanin biosynthesis genes across the four flowering stages. Two *CHS*, one *ANS*, and three *UGT* genes were screened. Red indicates gene upregulation, and green indicates downregulation.

**Figure 9:**
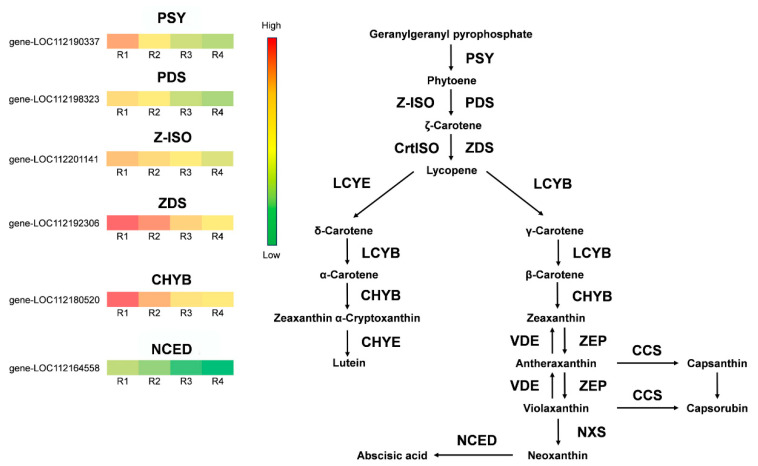
Expression patterns of key carotenoid biosynthesis genes across the four flowering stages. 1 *PSY*, 1 *PDS*, 1 *Z-ISO*, 1 *ZDS*, 1 *CHYB* and 1 *NCED* genes were screened out. Red indicates gene upregulation, and green indicates downregulation.

In this research, simplified heatmaps were created to depict the anthocyanin (Figure 8) and carotenoid (Figure 9) biosynthesis pathways. Based on a comprehensive transcriptome analysis of the rose cultivar ‘Rainbow’s End’, six genes were identified as differentially expressed, specifically within the context of the anthocyanin metabolic pathway. Specifically, the identified DEGs included two *CHS* genes, one *ANS* gene, and three *UGT* genes. These genes showed significant expression differences during R1-R4, with upregulation in R2 and R3 and downregulation in the R4 stage. The upstream gene *CHS* and the downstream genes *ANS* and *UGT* positively regulate anthocyanin synthesis in flower petals. *CHS* activity affects the intermediate products of anthocyanin synthesis. *ANS* is the key enzyme for the formation of color in colorless anthocyanins, and the *UGT* gene may enhance anthocyanin glycosylation, thereby stabilizing them. The high expression of these genes in R2 and R3 indicated large-scale synthesis of anthocyanins, which was consistent with the color changes in the flower petals.

Moreover, our analysis identified six critical genes in the carotenoid biosynthesis pathway, including one *PSY* gene, 1 *PDS* gene, 1 *Z-ISO* gene, 1 *ZDS* gene, 1 *CHYB* gene, and 1 *NCED* gene. These also showed expression differences during R1-R4; however, unlike anthocyanin pathway genes, their expression continuously decreased over this period. The reduced expression of these structural genes in R3 and R4 indicated low carotenoid biosynthesis, which was consistent with the fading of the flower petals’ yellow color.

### 3.7. Verifying DEGs Expression in the Transcriptome Data

**Figure 10:**
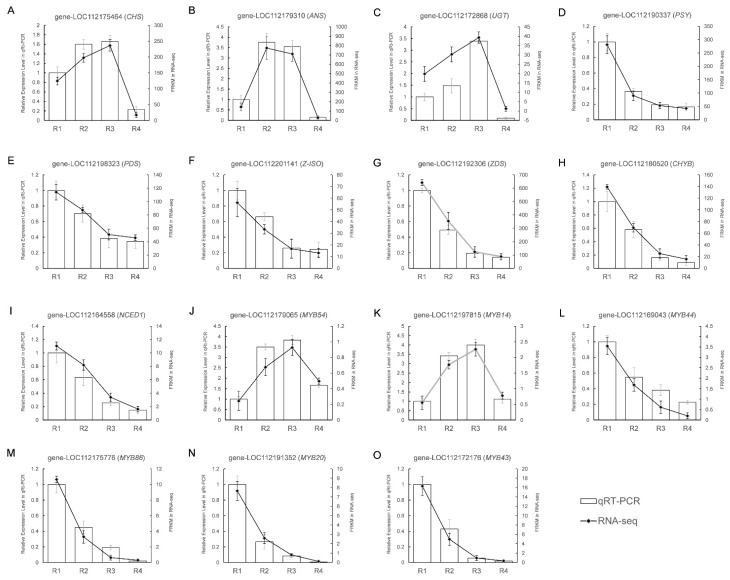
The changes in the expression levels of the 15 DEGs as determined by RNA-seq and qRT-PCR. (**A**–**C**) Genes in the anthocyanin biosynthetic pathway. (**D**–**I**) Genes in the carotenoid biosynthetic pathway. (**J**–**O**) Genes of MYB TFs. Error bars represent the standard deviations of the means.

We selected a subset of 15 genes with the most notable expression pattern fluctuations to verify the key findings regarding the differentially expressed genes, as illustrated further (Figure 10). This collection included three genes linked to the anthocyanin biosynthetic pathway, including *CHS* (gene-LOC112175464), *ANS* (gene-LOC112179310) and *UGT* (gene-LOC112172868), which were shown in Figure 10A–C; six genes involved in the carotenoid biosynthetic pathway, including *PSY* (gene-LOC112190337), *PDS* (gene-LOC112198323), *Z-ISO* (gene-LOC112201141), *ZDS* (gene-LOC112192306), *CHYB* (gene-LOC112180520) and *NCED* (gene-LOC112164558), which were shown in Figure 10D–I; and 6 MYB TFs, including *MYB44* (gene-LOC112169043), *MYB86* (gene-LOC112175776), *MYB20* (gene-LOC112175776), *MYB43* (gene-LOC112172176), *MYB54* (gene-LOC112179065), and *MYB14* (gene-LOC112197815), which were shown in Figure 10J–O. We analyzed the changes in gene expression across four flowering stages in Rosa ‘Rainbow’s End’ petals using quantitative reverse transcription polymerase chain reaction (qRT-PCR). The RNA-seq data closely matched the qRT-PCR results, demonstrating strong agreement in the patterns of upregulation and downregulation.

## 4. Discussion

The flower color of Rosa ‘Rainbow’s End’ shifts from yellow to red during blooming. This change is directly related to the production and buildup of anthocyanins and carotenoids during the flowering period. In this study, we divided the flowering stages of Rosa ‘Rainbow’s End’ into four stages based on color differences (Figure 1). Metabolomic analysis showed that anthocyanin levels were low during R1 and R2, reached their highest levels in R3, and then decreased in R4. In contrast, carotenoids mainly accumulated in stage R1, then declined in stage R2, and sharply decreased in stages R3 and R4 (Figure 3). Based on previous studies, cyanidin is usually closely associated with a red appearance [37]. Cyanidins play a role in color determination and are more common than other anthocyanidins in vegetables and fruits [38]. Targeted metabolomics analysis revealed that cyanidins were significantly more abundant than other anthocyanins, with their levels notably increasing during the R2 and R3 stages (Figure 2 and Table S1a–c). This result indicated that the red coloration that began at R2 was primarily due to the biosynthesis and accumulation of cyanidin. In contrast, the levels of most carotenoid metabolites continuously decreased from R1 to R4. This result was consistent with the yellow color fading of Rosa ‘Rainbow’s End’ petals.

KEGG enrichment analysis revealed that the color variation in Rosa ‘Rainbow’s End’ flowers was mainly concentrated in the anthocyanin and carotenoid biosynthesis pathways. Previous research by Liu et al. showed that a decrease in anthocyanin production combined with an increase in chlorophyll and carotenoid synthesis could explain the shift in the color of ornamental kale leaves from pink to a mixed pink-green color [39]. Similarly, Su et al. identified carotenoids as the main metabolites responsible for the orange coloration of the loquat cultivar Jiefangzhong, whereas cyanidin-3-O-galactoside was the dominant anthocyanin contributing to the red color of wild loquat fruits [40]. Huang et al. further showed that anthocyanins and carotenoids were key pigments in purple and yellow flowers, specifically in *Medicago sativa* ssp. [41]. These studies reinforce the notion that the genes associated with anthocyanin and carotenoid biosynthesis are vital for the color changes observed in Rosa Rainbow’s End flowers.

The biosynthesis and accumulation of anthocyanins and carotenoids depend on the expression of structural genes in these pathways, including *PAL*, *CHS*, *CHI*, *ANS*, *UGT*, *PSY*, *PDS*, *ZDS*, and *CHYB* [42,43]. Modulation of gene expression levels, whether through upregulation or downregulation, affects the synthesis and metabolic pathways of anthocyanins and carotenoids. Our metabolite and transcriptome analyses revealed that *CHS*, *ANS*, and *UGT* were highly expressed in R2 and R3 but were weakly expressed in R1 and R4. CHS catalyzes the first committed step in anthocyanin biosynthesis to form naringenin chalcone; ANS converts leucocyanidin into anthocyanidins, and UGT catalyzes the glycosylation of anthocyanidins to form anthocyanins [44,45]. They all play key roles in anthocyanin biosynthesis, and their expression patterns are consistent with changes in the amount of anthocyanins in Rosa Rainbow’s End petals (Figure 8). Therefore, we considered CHS, ANS, and UGT as key genes associated with the red coloration of Rosa Rainbow’s End. In contrast, *PSY*, *PDS*, *Z-ISO*, *ZDS*, *CHYB*, and *NCED* were highly expressed in R1, and their expression levels decreased continuously from R2 to R4. PSY is the first dedicated enzyme in the plant carotenoid biosynthesis pathway, and PDS, Z-ISO, ZDS, and CHYB also play important roles in the conversion of phytoene to violaxanthin [46]. The *NCED* gene is related to the breakdown of carotenoids and promotes ABA biosynthesis; however, some studies have also indicated that NCED plays a role in carotenoid-derived pigmentation, as evidenced in snapdragons, where NCED activity is linked to yellow pigmentation accumulation [47]. These genes had the same expression pattern as the changes in the amount of carotenoids; therefore, we consider them to be key genes that correlated with yellow color fading in Rosa ‘Rainbow’s End’ petals (Figure 9).

Various TFs, notably MYB, bHLH, and WD40, control anthocyanin synthesis in plants through transcriptional regulation by forming complexes to regulate anthocyanins [48]. The MYB family is large in plants and is divided into R3-, R2R3-, and R1R2R3-MYB based on their domains [49]. R2R3-MYB factors are vital for regulating both anthocyanin and carotenoid biosynthesis, with both positive and negative effects. Sagawa et al. found an R2R3-MYB that affects carotenoid pigmentation in Mimulus lewisii [50]. Wang et al. proved that MYB118 is important for anthocyanin biosynthesis in *Populus deltoids* [51]. Our study identified four MYB genes—*RcMYB20*, *RcMYB43, RcMYB44*, *and RcMYB86* —that were highly expressed during the flowering stage R1, decreased in R2, and remained low in R3 and R4, mirroring carotenoid biosynthesis gene expression (Figure 9). These MYBs may positively regulate carotenoid metabolism, contributing to the yellow coloration of Rosa ‘Rainbow’s End’. Conversely, *RcMYB14* and *RcMYB54* showed higher expression in R2 and R3, consistent with anthocyanin pathway genes (Figure 8), which may promote red coloration in Rosa ‘Rainbow’s End’ petals. Ye et al. found that MYB44 positively regulates carotenoid biosynthesis in *Brassica napus* [52], and Dong et al. reported that MYB14 interacts with PIF4-2 and positively regulates anthocyanin biosynthesis [53]. These reports support our conclusion. This study elucidates the mechanisms of color change during Rosa ‘Rainbow’s End’ flowering, aiding in flower breeding.

## 5. Conclusions

In this study, the *Rosa chinensis* cultivar, Rainbow’s End’ was used as the main experimental subject at four flowering stages. A detailed investigation of the discoloration mechanism during the flowering phase of this variety was conducted through a comprehensive integration of phenotypic, transcriptomic, and metabolomic analyses. Metabolomic profiling revealed that carotenoid levels increased during the initial flowering periodand declined in the mid- and late-stages. In contrast, anthocyanin concentrations showed a different pattern, starting at lower levels in the fully yellow stage, increasing in the yellow-orange and fully red stages, and decreasing in the white-pink stage. Transcriptome sequencing identified six genes involved in the anthocyanin biosynthetic pathway, comprising two *CHS* genes, one *ANS* gene, and three *UGT* genes. Concurrently, another set of six genes involved in carotenoid biosynthesis was identified, which included one *PSY* gene, one *PDS* gene, one *Z-ISO* gene, one *ZDS* gene, one *CHYB* gene, and one *NCEB* gene. These structural genes, along with two MYB transcription factors (MYB14 and MYB54) for the anthocyanin pathway and four (MYB20, MYB43, MYB44, and MYB86) for the carotenoid pathway, were connected to the color changes observed in the flowers of Rosa ‘Rainbow’s End’. Overall, this research highlights the key roles of specific anthocyanins, carotenoids, and related genes in flower pigmentation in Rosa ‘Rainbow’s End’, laying a foundation for future studies.

## Figures and Tables

**Table 1:** Ratios of each anthocyanidin and carotenoid metabolite class in the total amount during the four flowering stages of Rosa ‘Rainbow’s End’.

Metabolites Class	R1	R2	R3	R4
Cyanidin	13.71%	36.41%	72.03%	83.69%
Delphinidin	0.70%	0.40%	0.48%	0.46%
Malvidin	0.02%	0.01%	0.01%	0.01%
Pelargonidin	2.33%	8.11%	10.65%	12.19%
Peonidin	0.13%	0.17%	0.64%	0.59%
Petunidin	N/A	N/A	0.01%	0.01%
Carotenes	5.31%	1.90%	0.37%	0.25%
Xanthophylls	77.80%	53.00%	15.81%	2.80%

R1: Fully yellow flowers; R2: Yellow-orange flowers; R3: Fully red flowers; R4: White-pink flowers. N/A: Cannot be detected in the sample.

## Data Availability

All data are open and available. The raw data are available in the Genome Sequence Archive (Genomics, Proteomics & Bioinformatics 2021) in the National Genomics Data Center (Nucleic Acids Res 2021), China National Center for Bioinformation/Beijing Institute of Genomics, Chinese Academy of Sciences (GSA: CRA035449).

## Supplementary Materials

The following supporting information can be downloaded at: https://www.mdpi.com/article/10.3390/metabo16010032/s1, Table S1: Anthocyanidin DEMs detected in (a) R2 vs. R1 (b) R3 vs. R2, (c) R4 vs. R3; Table S2: Carotenoid DEMs detected in (a) R2 vs. R1 (b) R3 vs. R2 (c) R4 vs. R3; Table S3: Anthocyanidin DEMs detected in (a) R3 vs. R1 (b) R4 vs. R1 (c) R4 vs. R2; Table S4: Carotenoid DEMs detected in (a) R3 vs. R1 (b) R4 vs. R1 (c) R4 vs. R2; Table S5: Summary of sequencing data quality; Table S6: Primers used for real-time quantitative RT-PCR.

## References

[1] Noman A., Aqeel M., Deng J., Khalid N., Sanaullah T., Shuilin H. (2017). Biotechnological Advancements for Improving Floral Attributes in Ornamental Plants. Front. Plant Sci..

[2] Marin-Recinos M.F., Pucker B. (2024). Genetic factors explaining anthocyanin pigmentation differences. BMC Plant Biol..

[3] Brugliera F., Tao G.Q., Tems U., Kalc G., Mouradova E., Price K., Stevenson K., Nakamura N., Stacey I., Katsumoto Y. (2013). Violet/blue chrysanthemums—metabolic engineering of the anthocyanin biosynthetic pathway results in novel petal colors. Plant Cell Physiol..

[4] Ye L.J., Möller M., Luo Y.H., Zou J.Y., Zheng W., Wang Y.H., Liu J., Zhu A.D., Hu J.Y., Li D.Z. (2021). Differential expressions of anthocyanin synthesis genes underlie flower color divergence in a sympatric Rhododendron sanguineum complex. BMC Plant Biol..

[5] Ho W.W., Smith S.D. (2016). Molecular evolution of anthocyanin pigmentation genes following losses of flower color. BMC Evol. Biol..

[6] Sun T., Rao S., Zhou X., Li L. (2022). Plant carotenoids: Recent advances and future perspectives. Mol. Hortic..

[7] Isaacson T., Ronen G., Zamir D., Hirschberg J. (2002). Cloning of tangerine from tomato reveals a carotenoid isomerase essential for the production of beta-carotene and xanthophylls in plants. Plant Cell.

[8] Liu W., Zheng T., Yang Y., Li P., Qiu L., Li L., Wang J., Cheng T., Zhang Q. (2021). Meta-Analysis of the Effect of Overexpression of MYB Transcription Factors on the Regulatory Mechanisms of Anthocyanin Biosynthesis. Front. Plant Sci..

[9] Chen L., Cui Y., Yao Y., An L., Bai Y., Li X., Yao X., Wu K. (2023). Genome-wide identification of WD40 transcription factors and their regulation of the MYB-bHLH-WD40 (MBW) complex related to anthocyanin synthesis in Qingke (*Hordeum vulgare*, L. var. nudum Hook. f.). BMC Genom..

[10] Yang T., Wang Y., Li Y., Liang S., Yang Y., Huang Z., Li Y., Gao J., Ma N., Zhou X. (2024). The transcription factor RhMYB17 regulates the homeotic transformation of floral organs in rose (*Rosa hybrida*) under cold stress. J. Exp. Bot..

[11] Xie G., Zou X., Liang Z., Wu D., He J., Xie K., Jin H., Wang H., Shen Q. (2022). Integrated metabolomic and transcriptomic analyses reveal molecular response of anthocyanins biosynthesis in perilla to light intensity. Front. Plant Sci..

[12] Liu Y., Li Y., Liu Z., Wang L., Lin-Wang K., Zhu J., Bi Z., Sun C., Zhang J., Bai J. (2023). Integrative analysis of metabolome and transcriptome reveals a dynamic regulatory network of potato tuber pigmentation. iScience.

[13] Albert N.W., Davies K.M., Lewis D.H., Zhang H., Montefiori M., Brendolise C., Boase M.R., Ngo H., Jameson P.E., Schwinn K.E. (2014). A conserved network of transcriptional activators and repressors regulates anthocyanin pigmentation in eudicots. Plant Cell.

[14] He G., Zhang R., Jiang S., Wang H., Ming F. (2023). The MYB transcription factor RcMYB1 plays a central role in rose anthocyanin biosynthesis. Hortic. Res..

[15] Ma S., Zhou H., Ren T., Yu E.R., Feng B., Wang J., Zhang C., Zhou C., Li Y. (2024). Integrated transcriptome and metabolome analysis revealed that HaMYB1 modulates anthocyanin accumulation to deepen sunflower flower color. Plant Cell Rep..

[16] Kishimoto S., Ohmiya A. (2012). Carotenoid isomerase is key determinant of petal color of Calendula officinalis. J. Biol. Chem..

[17] Kljak K., Carovic-Stanko K., Kos I., Janjecic Z., Kis G., Duvnjak M., Safner T., Bedekovic D. (2021). Plant Carotenoids as Pigment Sources in Laying Hen Diets: Effect on Yolk Color, Carotenoid Content, Oxidative Stability and Sensory Properties of Eggs. Foods.

[18] Zhang H., Zhang S., Zhang H., Chen X., Liang F., Qin H., Zhang Y., Cong R., Xin H., Zhang Z. (2020). Carotenoid metabolite and transcriptome dynamics underlying flower color in marigold (*Tagetes erecta* L.). Sci. Rep..

[19] Fan Y., Sun L., Song S., Sun Y., Fan X., Li Y., Li R., Sun H. (2023). Integrated metabolome and transcriptome analysis of anthocyanin accumulation during the color formation of bicolor flowers in Eustoma grandiflorum. Sci. Hortic..

[20] Lee J.M., Joung J.G., McQuinn R., Chung M.Y., Fei Z., Tieman D., Klee H., Giovannoni J. (2012). Combined transcriptome, genetic diversity and metabolite profiling in tomato fruit reveals that the ethylene response factor SlERF6 plays an important role in ripening and carotenoid accumulation. Plant J..

[21] Hussain Q., Asim M., Zhang R., Khan R., Farooq S., Wu J. (2021). Transcription Factors Interact with ABA through Gene Expression and Signaling Pathways to Mitigate Drought and Salinity Stress. Biomolecules.

[22] Lim C., Kang K., Shim Y., Yoo S.C., Paek N.C. (2022). Inactivating transcription factor OsWRKY5 enhances drought tolerance through abscisic acid signaling pathways. Plant Physiol..

[23] Singh D., Debnath P., Roohi Sane A.P., Sane V.A. (2020). Expression of the tomato WRKY gene, SlWRKY23, alters root sensitivity to ethylene, auxin and JA and affects aerial architecture in transgenic Arabidopsis. Physiol. Mol. Biol. Plants.

[24] Xu G., Huang J., Lei S.K., Sun X.G., Li X. (2019). Comparative gene expression profile analysis of ovules provides insights into Jatropha curcas, L. ovule development. Sci. Rep..

[25] Wu L., Wang K., Chen M., Su W., Liu Z., Guo X., Ma M., Qian S., Deng Y., Wang H. (2024). ALLENE OXIDE SYNTHASE (AOS) induces petal senescence through a novel JA-associated regulatory pathway in Arabidopsis. Physiol. Mol. Biol. Plants.

[26] Lee K.Y., Shin J.Y., Ahn M.S., Kim S.J., An H.R., Kim Y.J., Kwon O.H., Lee S.Y. (2023). Callus Derived from Petals of the *Rosa hybrida* Breeding Line 15R-12-2 as New Material Useful for Fragrance Production. Plants.

[27] Simin N., Zivanovic N., Bozanic Tanjga B., Lesjak M., Narandzic T., Ljubojevic M. (2024). New Garden Rose (*Rosa x hybrida*) Genotypes with Intensely Colored Flowers as Rich Sources of Bioactive Compounds. Plants.

[28] Xuan Y., Ren J., Chen Z., Shi D. (2025). Integrated transcriptome and metabolome analyses provide molecular insights into the transition of flower color in the rose cultivar ‘Juicy Terrazza’. BMC Plant Biol..

[29] Deng X., Hu C., Xie C., Lu A., Luo Y., Peng T., Huang W. (2023). Metabolomic and Transcriptomic Analysis Reveal the Role of Metabolites and Genes in Modulating Flower Color of *Paphiopedilum micranthum*. Plants.

[30] Li B.-J., Zheng B.-Q., Wang J.-Y., Tsai W.-C., Lu H.-C., Zou L.-H., Wan X., Zhang D.-Y., Qiao H.-J., Liu Z.-J. (2020). New insight into the molecular mechanism of colour differentiation among floral segments in orchids. Commun. Biol..

[31] Geyer R., Peacock A.D., White D.C., Lytle C., Van Berkel G.J. (2004). Atmospheric pressure chemical ionization and atmospheric pressure photoionization for simultaneous mass spectrometric analysis of microbial respiratory ubiquinones and menaquinones. J. Mass. Spectrom..

[32] de Ferrars R.M., Czank C., Saha S., Needs P.W., Zhang Q., Raheem K.S., Botting N.P., Kroon P.A., Kay C.D. (2014). Methods for isolating, identifying, and quantifying anthocyanin metabolites in clinical samples. Anal. Chem..

[33] Kim D., Langmead B., Salzberg S.L. (2015). HISAT: A fast spliced aligner with low memory requirements. Nat. Methods.

[34] Pertea M., Pertea G.M., Antonescu C.M., Chang T.C., Mendell J.T., Salzberg S.L. (2015). StringTie enables improved reconstruction of a transcriptome from RNA-seq reads. Nat. Biotechnol..

[35] Trapnell C., Williams B.A., Pertea G., Mortazavi A., Kwan G., van Baren M.J., Salzberg S.L., Wold B.J., Pachter L. (2010). Transcript assembly and quantification by RNA Seq reveals unannotated transcripts and isoform switching during cell differentiation. Nat. Biotechnol..

[36] Love M.I., Huber W., Anders S. (2014). Moderated estimation of fold change and dispersion for RNA-seq data with DESeq2. Genome Biol..

[37] Park C.H., Yeo H.J., Kim N.S., Park Y.E., Park S.Y., Kim J.K., Park S.U. (2018). Metabolomic Profiling of the White, Violet, and Red Flowers of *Rhododendron schlippenbachii* Maxim. Molecules.

[38] Kong J.-M., Chia L.-S., Goh N.-K., Chia T.-F., Brouillard R. (2008). Corrigendum to “Analysis and biological activities of anthocyanins” [Phytochemistry 64 (2003) 923–933]. Phytochemistry.

[39] Liu Y., Feng X., Zhang Y., Zhou F., Zhu P. (2021). Simultaneous changes in anthocyanin, chlorophyll, and carotenoid contents produce green variegation in pink-leaved ornamental kale. BMC Genom..

[40] Su W., Zhu C., Fan Z., Huang M., Lin H., Chen X., Deng C., Chen Y., Kou Y., Tong Z. (2023). Comprehensive metabolome and transcriptome analyses demonstrate divergent anthocyanin and carotenoid accumulation in fruits of wild and cultivated loquats. Front. Plant Sci..

[41] Huang X., Liu L., Qiang X., Meng Y., Li Z., Huang F. (2024). Integrated Metabolomic and Transcriptomic Profiles Provide Insights into the Mechanisms of Anthocyanin and Carotenoid Biosynthesis in Petals of Medicago sativa ssp. sativa and Medicago sativa ssp. falcata. Plants.

[42] Ma Y., Ma X., Gao X., Wu W., Zhou B. (2021). Light Induced Regulation Pathway of Anthocyanin Biosynthesis in Plants. Int. J. Mol. Sci..

[43] Quian-Ulloa R., Stange C. (2021). Carotenoid Biosynthesis and Plastid Development in Plants: The Role of Light. Int. J. Mol. Sci..

[44] Takos A.M., Jaffe F.W., Jacob S.R., Bogs J., Robinson S.P., Walker A.R. (2006). Light-induced expression of a MYB gene regulates anthocyanin biosynthesis in red apples. Plant Physiol..

[45] Qi Y., Gu C., Wang X., Gao S., Li C., Zhao C., Li C., Ma C., Zhang Q. (2020). Identification of the Eutrema salsugineum EsMYB90 gene important for anthocyanin biosynthesis. BMC Plant Biol..

[46] Giuliano G., Bartley G.E., Scolnik P.A. (1993). Regulation of carotenoid biosynthesis during tomato development. Plant Cell.

[47] Han Y., Cui Y., Chen Y., Rao D., Wu E., Gan R., Li T., Tian M. (2025). High-density genetic map construction using whole-genome resequencing of the *Cymbidium eburneum* (‘Duzhan Chun’) x *Cymbidium insigne* (‘Meihua Lan’) F1 population and localization of flower color genes. Front. Plant Sci..

[48] Sun X., Zhang Z., Li J., Zhang H., Peng Y., Li Z. (2022). Uncovering Hierarchical Regulation among MYB-bHLH-WD40 Proteins and Manipulating Anthocyanin Pigmentation in Rice. Int. J. Mol. Sci..

[49] Yang J., Zhang B., Gu G., Yuan J., Shen S., Jin L., Lin Z., Lin J., Xie X. (2022). Genome-wide identification and expression analysis of the R2R3-MYB gene family in tobacco (*Nicotiana tabacum* L.). BMC Genom..

[50] Sagawa J.M., Stanley L.E., LaFountain A.M., Frank H.A., Liu C., Yuan Y.W. (2016). An R2R3-MYB transcription factor regulates carotenoid pigmentation in *Mimulus lewisii* flowers. New Phytol..

[51] Wang H., Wang X., Yu C., Wang C., Jin Y., Zhang H. (2020). MYB transcription factor PdMYB118 directly interacts with bHLH transcription factor PdTT8 to regulate wound-induced anthocyanin biosynthesis in poplar. BMC Plant Biol..

[52] Ye S., Huang Y., Ma T., Ma X., Li R., Shen J., Wen J. (2024). BnaABF3 and BnaMYB44 regulate the transcription of zeaxanthin epoxidase genes in carotenoid and abscisic acid biosynthesis. Plant Physiol..

[53] Dong X.M., Zhang W., Tu M., Zhang S.B. (2025). Spatial and Temporal Regulation of Flower Coloration in *Cymbidium lowianum*. Plant Cell Environ..

